# Global Research Trends in Irritable Bowel Syndrome: A Bibliometric and Visualized Study

**DOI:** 10.3389/fmed.2022.922063

**Published:** 2022-06-27

**Authors:** Tai Zhang, Xiangxue Ma, Wende Tian, Jiaqi Zhang, Yuchen Wei, Beihua Zhang, Fengyun Wang, Xudong Tang

**Affiliations:** ^1^Xiyuan Hospital, China Academy of Chinese Medical Sciences, Beijing, China; ^2^Department of Gastroenterology, Xiyuan Hospital, China Academy of Traditional Chinese Medical Sciences, Beijing, China; ^3^National Clinical Research Center for Chinese Medicine Cardiology, Xiyuan Hospital, China Academy of Chinese Medical Sciences, Beijing, China; ^4^Xiyuan Hospital, Traditional Chinese Medicine Research Institute of Spleen and Stomach Diseases, China Academy of Chinese Medical Sciences, Beijing, China

**Keywords:** irritable bowel syndrome, VOSviewer, CiteSpace, bibliometrics, hot topics, trends

## Abstract

**Background:**

There are about 10–23% of adults worldwide suffering from irritable bowel syndrome (IBS). Over the past few decades, there are many aspects of uncertainty regarding IBS leading to an ongoing interest in the topic as reflected by a vast number of publications, whose heterogeneity and variable quality may challenge researchers to measure their scientific impact, to identify collaborative networks, and to grasp actively researched themes. Accordingly, with help from bibliometric approaches, our goal is to assess the structure, evolution, and trends of IBS research between 2007 and 2022.

**Methods:**

The documents exclusively focusing on IBS from 2007 to 2022 were retrieved from the Science Citation Index Expanded of the Web of Science Core Collection. The annual productivity of IBS research, and the most prolific countries or regions, authors, journals and resource-, intellectual- and knowledge-sharing in IBS research, as well as co-citation analysis of references and keywords were analyzed through Microsoft Office Excel 2019, CiteSpace, and VOSviewer.

**Results:**

In total, 4,092 publications were reviewed. The USA led the list of countries with the most publications (1,226, 29.96%). Mayo Clinic contributed more publications than any other institution (193, 4.71%). MAGNUS SIMREN stood out as the most active and impactful scholar with the highest number of publications and the greatest betweenness centrality value. The most high-yield journal in this field was *Neurogastroenterology and motility: the official journal of the European Gastrointestinal Motility Society* (275, 6.72%). *Gastroenterology* had the most co-citations (3,721, 3.60%). Keywords with the ongoing strong citation bursts were chromogranin A, rat model, peptide YY, gut microbiota, and low-FODMAP diet, etc.

**Conclusion:**

Through bibliometric analysis, we gleaned deep insight into the current status of literature investigating IBS for the first time. These findings will be useful to scholars interested in understanding the key information in the field, as well as identifying possible research frontiers.

## Introduction

Associated with abdominal pain, bloating, and altered bowel habits, irritable bowel syndrome (IBS) is a chronic, cyclical and relapsing functional bowel disorder (1). The global prevalence of IBS is currently estimated at 15%, and IBS symptoms occur in about 10–20% of Westerners (2–4). Irrespective of bowel habit, diagnoses of IBS have traditionally been made by using the Rome diagnostic criteria, which is a symptom-based diagnostic standard that is being updated to Rome IV criteria (5). Further subtypes of IBS include diarrhea-predominant IBS (IBS-D), constipation-predominant IBS (IBS-C), mixed type of IBS with both diarrhea and constipation (IBS-M), and unclassified IBS (6, 7). A key challenge that has faced IBS research to date has been the pathophysiology, which is thought to be multifactorial (8). There are still no satisfactory treatments for patients with IBS because of its complex pathogenesis. Currently, there is an emphasis on symptomatic management; yet, it involves multiple medications and fails to address the underlying complex pathogenesis of the disease, with approximately one-third of patients failing to respond (9–12).

As a result of the multiple and persistent symptoms of IBS, it contributes to a decline in quality of life, high absenteeism, and high socioeconomic burden. It has been estimated that between 8.5 and 21.6 days a year are taken off work due to IBS. There are approximately 3.6 million physician office visits related to IBS every year, resulting in healthcare costs of more than $30 billion (13–15).

There are many aspects of uncertainty regarding IBS, leading to an ongoing interest in the field as reflected by the huge amount of literature. Thus, it is difficult to characterize the evolution of knowledge components, the current body of knowledge, and the research trends.

The bibliometric analysis utilizes mathematical and statistical methods and involves the use of a series of defined metrics to evaluate the structure, productivity, progress, quality, impact and inter-connectivity of scientific work (16, 17). One way to accurately capture and integrate data from disparate sources of heterogeneous information is through a knowledge map, which visualizes the connections between complex data silos (18). Furthermore, key authors, institutions and countries as well as the structure of scientific collaboration networks can be identified. However, there are few bibliometric studies on IBS research. In this context, the present study aims to use a bibliometric approach to identify, evaluate and visualize all literature published on IBS since 2007 regarding quantitative, semiqualitative, and chronological elements of data collected.

## Materials and Methods

### Source of the Data and Search Strategy

The search was performed on the Science Citation Index Expanded of the Web of Science Core Collection (WoSCC) of Clarivate Analytics. All searches were conducted on the same day, February 1, 2022. The literature search was completed by two authors independently for identifying IBS-related publications with the following search strategy: TOPIC:[(adaptive colitis) OR (colon spasm) OR (functional bowel disease) OR (irritable bowel) OR (irritable colon) OR (membranous colitis) OR (mucous colitis) OR (spastic colitis) OR (spastic colon) OR (spastic bowel) OR (functional colonic disease) OR (colon irritable) OR (colon neurosis) OR (bowel neurosis) OR (functional colopathy) OR (functional colonopathy) OR (chronic catarrhal colitis) OR (colica mucosa) OR (colonic enterospasm) OR (dyskinesia of the colon) OR (dyssynergia of the colon) OR (functional enterocolonopathy) OR (Glarry enteritis) OR (glutinous diarrhea) OR (intestinal croup) OR (irritable gut syndrome) OR (lienteric diarrhea) OR (membranous catarrh of the intestine) OR (mucomembranous colic) OR (myxoneurosis) OR (nervous diarrhea) OR (neurogenic mucous) OR (non-specific diarrhea) OR (tubular diarrhea) OR (unhappy colon) OR (unstable colon)] AND Language:(English). Additionally, articles and reviews containing at least one search term in the “title” were included since the aim was to obtain the academic research on the topic of interest. However, the “TOPIC” search enables the inclusion of a considerable amount of off-topic publications with the search terms in abstract, author keywords and keywords plus. The retrieval time was from February 1, 2007 until February 1, 2022. The bibliographic records were collected and saved in plain text. Ultimately, these documents were imported into CiteSpace and VOSviewer for analysis.

### Data Analysis

CiteSpace (19), which is a freely available Java-based software package developed by Professor Chaomei Chen at Drexel University, was applied to (1) perform co-occurrence analysis; (2) visualize key features of literature, such as authors, countries or regions, organizations, and keywords; (3) perform a co-citation analysis of references; (4) depict timeline view of keywords; and (5) capture keywords and references with strong citation bursts. VOSviewer (20), which is a free software tool based on the Java environment developed by Nees Jan van Eck and Ludo Waltman from Leiden University, was used for creating clusters of keywords. Microsoft Excel 2019 was used to demonstrate the amount of scientific literature published annually.

## Results

### Publication Output

**FIGURE 1:**
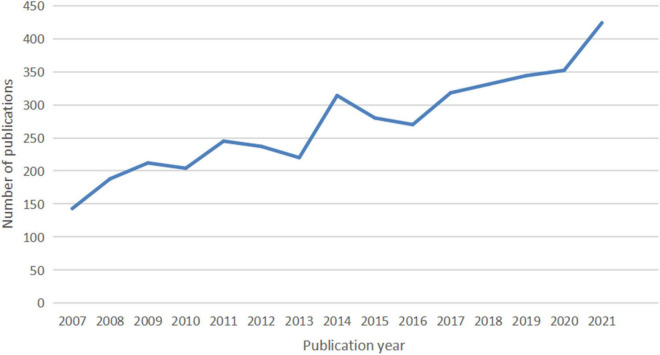
The number of articles published annually in IBS research.

A total of 4,092 publications were identified, including 3,299 articles (80.62%) and 793 reviews (19.37%). The number of publications per year since 2007 is shown in Figure 1. There is an overall trend of increased output of scientific research over time, which falls into two stages. The first phase from 2007 to 2016 exhibited a growing trend despite the decrease in 2010, 2013, 2015, and 2016. As the second stage has progressed, the number of documents has increased from 270 in 2016 to 344 in 2019, to 424 in 2021. The volume of papers published during the last 6 years (2016–2021) accounted for 49.82% of all publications.

### Countries or Regions and Institutions Analysis

In total, IBS articles were published by 407 institutions from 116 countries or regions. As shown in Table 1, the top 10 countries and institutions are listed. The United Stateswas the leading country in the field, which had an overwhelmingly higher number of publications (1,226, 29.96%). China ranked second (577, 14.10%). In third place is England (421, 10.28%).

**TABLE 1:** The top 10 countries or regions and institutions involved in IBS research.

Rank	Country	Centrality	Count (% of 4,092)	Institutions	Centrality	Count (% of 4,092)
1	United States	0.54	1,226 (29.96)	Mayo Clin (United States)	0.11	193 (4.71)
2	PEOPLES R CHINA	0.01	577 (14.10)	Univ Calif Los Angeles (United States)	0.05	142 (3.47)
3	ENGLAND	0.16	421 (10.28)	Univ N Carolina (United States)	0.1	122 (2.98)
4	SWEDEN	0.12	230 (5.62)	Univ Gothenburg (Sweden)	0.15	114 (2.78)
5	ITALY	0.04	222 (5.42)	Univ Washington (United States)	0.12	95 (2.32)
6	AUSTRALIA	0.02	206 (6.82)	Univ Bergen (Norway)	0.02	77 (1.88)
7	GERMANY	0.1	192 (5.03)	McMaster Univ (Canada)	0.09	70 (1.71)
8	CANADA	0.24	177 (4.32)	Univ Leeds (England)	0.07	66 (1.61)
9	SOUTH KOREA	0.03	154 (3.76)	Univ Tehran Med Sci (Iran)	0.07	64 (1.56)
10	FRANCE	0.1	134 (3.27)	Kings Coll London (England)	0.03	59 (1.44)
10	JAPAN	0.04	134 (3.27)			

With regard to contributions of institutions, the majority of the top 10 prolific institutions were from the USA (40%) and England (20%). Among them, Mayo Clin contributed the most publications (193, 4.71%), followed by Univ Calif Los Angeles (142, 3.47%) and Univ N Carolina (122, 2.98%).

In addition, the USA ranked first by the betweenness centrality value (0.54), followed by Canada (0.24), and England (0.16). Univ Gothenburg ranked first by the betweenness centrality value (0.15), followed by Univ Washington (0.12) and Mayo Clin (0.11).

**FIGURE 2:**
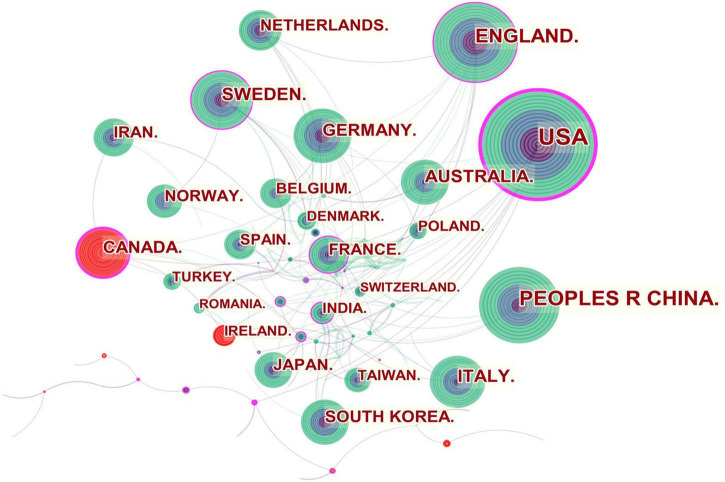
Network of countries and regions engaged in IBS research.

Figure 2 shows the collaboration among the countries or regions. In the map, each node represents a country or territory. The radius of a node increases with its contribution to the research on IBS. The links between nodes represent the collaboration, whereby their thicknesses are proportional to the intensity of the collaboration. A node’s betweenness centrality is calculated in order to identify the node that lies between two or more large groups of nodes. In a network, a node with a betweenness centrality value of more than 0.1 (i.e., one interconnected with more than 10% of the other nodes) exerts substantial influence over others because more information passes through that node. A node with a high betweenness centrality value is marked with a purple ring, while a red ring denotes a burst.

The United States, Canada, England, Sweden, France and India were referred to as central countries for the network owing to their cooperation occurring worldwide. For example, the United States, which possessed the broadest scientific collaboration, worked intensively with Australia, Peru, Israel, Sweden, Canada, Netherlands, Russia, Japan, and South Korea. Canada had close cooperation with Iran, England, the United States, South Africa, France, Argentina, Mexico, and Ireland. The main collaborators with England were the Netherlands, Germany, Australia, New Zealand, Scotland, Jordan, Palestine, Pakistan and Switzerland. Strong bursts were detected for Canada and Ireland.

**FIGURE 3:**
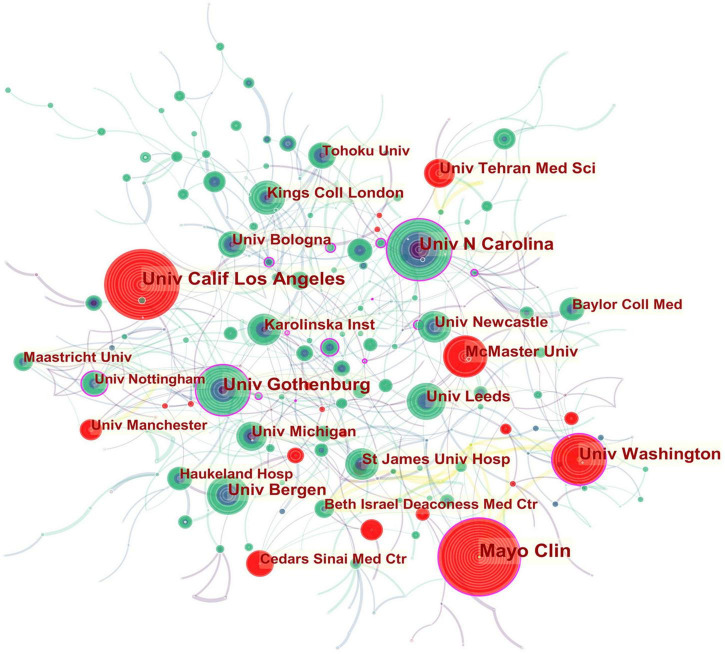
Network of institutions engaged in IBS research.

In the institutional collaboration network shown in Figure 3, the landmark nodes included Univ Gothenburg, Univ Washington, Mayo Clin, Univ N Carolina, and Univ Nottingham, signifying that they partnered extensively with academic organizations across the globe. The main institutions that collaborated with Univ Gothenburg were Karolinska Inst, Univ N Carolina, Univ North Carolina Chapel Hill, Univ Copenhagen, Sahlgrens Univ Hosp, Sabbatsbergs Hosp, AstraZeneca R&D, Katholieke Univ Leuven, and Univ Leuven. Univ Washington collaborated actively with Keimyung Univ, Ewha Womans Univ, Fred Hutchinson Canc Res Ctr, Broad Inst MIT and Harvard, Harvard Med Sch, Brigham and Womens Hosp, Mayo Clin, Grp Hlth Cooperat Puget Sound, and Campbell Univ. Mayo Clin cooperated frequently with Harvard Med Sch, Baylor Coll Med, Univ Complutense, Univ Sydney, Broad Inst MIT and Harvard, Univ Washington, Montefiore Med Ctr, and Brigham and Womens Hosp. Teheran Univ Med Sci, Univ Calif Los Angeles, Mayo Clin, Univ Washington, McMaster Univ, Univ Manchester, and Cedars Sinai Med Ctr were detected with strong bursts.

### Authors

In all, 423 authors contributed to the IBS studies. As shown in Table 2, IBS articles were mostly published by authors affiliated with institutions in America (308). MAGNUS SIMREN contributed the most articles (87, 2.12%), followed by MICHAEL CAMILLERI (75, 1.83%), and ALEXANDER C FORD (68, 1.66%). The top authors by the betweenness centrality value were MAGNUS SIMREN (0.13), EMERAN A MAYER (0.12), MICHAEL CAMILLERI (0.09), and NICHOLAS J TALLEY (0.09).

**TABLE 2:** The top 10 authors of IBS research.

Rank	Author	Count (% of 4,092)	Centrality
1	MAGNUS SIMREN (Sweden)	87 (2.12)	0.13
2	MICHAEL CAMILLERI (the United States)	75 (1.83)	0.09
3	ALEXANDER C FORD (England)	68 (1.66)	0.07
4	NICHOLAS J TALLEY (Australia)	65 (1.58)	0.09
5	EAMONN M M QUIGLEY (the United States)	55 (1.34)	0.04
6	WILLIAM D CHEY (the United States)	50 (1.22)	0.07
7	EMERAN A MAYER (the United States)	48 (1.17)	0.12
8	MAGDY ELSALHY (Norway)	47 (1.14)	0
8	HANS TORNBLOM (Sweden)	47 (1.14)	0
9	LIN CHANG (the United States)	41 (1.00)	0.01
10	MARK PIMENTEL (the United States)	39 (0.95)	0.02

**FIGURE 4:**
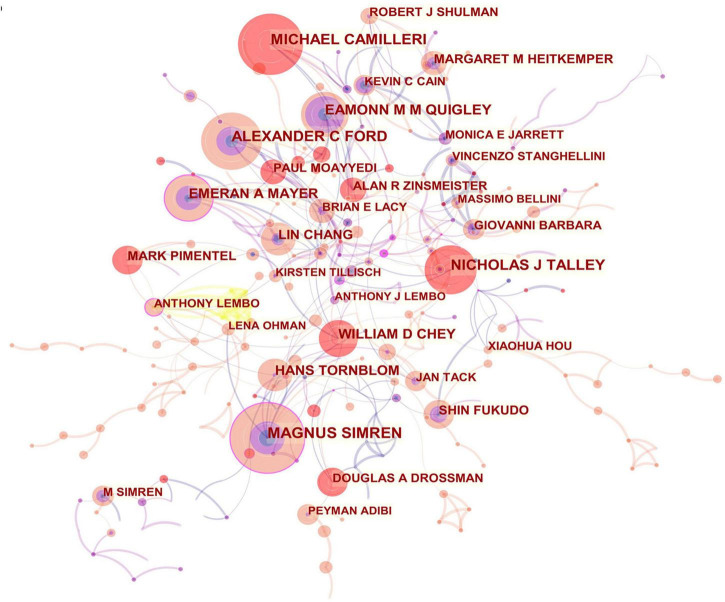
Network of authors in IBS research.

From the author’s collaboration network, which is presented in Figure 4, MAGNUS SIMREN and EMERAN A MAYER were located at a central position in the collaboration network. Active collaborations were seen among MAGNUS SIMREN, GUY BOECKXSTAENS (Belgium), LENA OHMAN (Sweden), GISELA RINGSTROM (Sweden), IRIS POSSERUD (Sweden), HANS TORNBLOM (Sweden), EVA JAKOBSSON UNG (Sweden), STINE STORSRUD (Sweden), OLAFUR S PALSSON (the United States), and HASSE ABRAHAMSSON (Sweden). EMERAN A MAYER had close communication with BEATE NIESLER (Germany), BRUCE NALIBOFF (the United States), WENDY SHIH (the United States), ANGELA P PRESSON (Germany), ARPANA GUPTA (the United States), JENNIFER S LABUS (the United States), GUY BOECKXSTAENS (Belgium), and KIRSTEN TILLISCH (the United States).

### Journals and Co-cited Academic Journals

Publications pertaining to IBS research were found in 799 journals. Below is a brief summary of the 10 most prolific journals as shown in Table 3. Among them, *Neurogastroenterology and motility: the official journal of the European Gastrointestinal Motility Society* published the highest number of articles (275, 6.72%), followed by *World journal of gastroenterology* (171, 4.17%), and *Alimentary pharmacology and therapeutics* (166, 4.05%). *Gastroenterology* with the highest impact factor (IF) of 22.682, published 76 articles (1.85%), ranked ninth for the total number of scientific articles. While the journal with the lowest IF of 3.067 was *BMC gastroenterology*, which ranked eighth with 78 articles (1.90%).

**TABLE 3:** Top 10 journal and top 10 co-cited journals in IBS research.

Rank	Journal	Count (% of 4,092)	IF	JCR	Rank	Co-cited journal	Count (% of 103,309)	IF	JCR
1	NEUROGASTROENTEROLOGY AND MOTILITY (England)	275 (6.72)	3.598	Q3	1	GASTROENTEROLOGY (the United States)	3,721 (3.60)	22.682	Q1
2	WORLD JOURNAL OF GASTROENTEROLOGY (the United States)	171 (4.17)	5.742	Q2	2	AMERICAN JOURNAL OF GASTROENTEROLOGY (the United States)	3,350 (3.24)	10.864	Q1
3	ALIMENTARY PHARMACOLOGY AND THERAPEUTICS (England)	166 (4.05)	8.171	Q1	3	GUT (the United SA)	3,155 (3.05)	23.059	Q1
4	AMERICAN JOURNAL OF GASTROENTEROLOGY (the United States)	142 (3.47)	10.864	Q1	4	ALIMENTARY PHARMACOLOGY AND THERAPEUTICS (England)	2,802 (2.71)	8.171	Q1
5	JOURNAL OF NEUROGASTROENTEROLOGY AND MOTILITY (South Korea)	101 (2.46)	4.924	Q1	5	NEUROGASTROENTEROLOGY AND MOTILITY (England)	2,472 (2.39)	3.598	Q3
6	CLINICAL GASTROENTEROLOGY AND HEPATOLOGY (the United States)	91 (2.22)	11.382	Q1	6	DIGESTIVE DISEASES AND SCIENCES (the United States)	2,185 (2.11)	3.199	Q3
7	DIGESTIVE DISEASES AND SCIENCES (the United States)	85 (2.07)	3.199	Q3	7	CLINICAL GASTROENTEROLOGY AND HEPATOLOGY (the United States)	2,057 (1.99)	11.382	Q1
8	BMC GASTROENTEROLOGY (England)	78 (1.90)	3.067	Q3	8	WORLD JOURNAL OF GASTROENTEROLOGY (the United States)	1,835 (1.77)	5.742	Q2
9	PLOS ONE (the United States)	76 (1.85)	3.24	Q3	9	SCANDINA‘N JOURNAL OF GASTROENTEROLOGY (England)	1,605 (1.55)	2.423	Q4
9	GASTROENTEROLOGY (the United States)	76 (1.85)	22.682	Q1	10	JOURNAL OF GASTROENTEROLOGY AND HEPATOLOGY (Australia)	1,263 (1.22)	4.029	Q2
10	JOURNAL OF GASTROENTEROLOGY AND HEPATOLOGY (Australia)	70 (1.71)	4.029	Q2					

When two or more documents are cited simultaneously by a third paper, the former is termed as “co-cited” (21). Due to the scientific and objective nature of the co-citation analysis, subjects have been expanded from papers to authors, journals, and disciplines. The frequency at which the documents of two journals are cited together by the documents of another journal is called journal co-citation (22, 23). The papers that published research in IBS were co-cited by 1,394 scholarly journals. As shown in Table 3, *Gastroenterology* had the most co-citations (3,721, 3.60%), followed by *The American journal of gastroenterology* (3,350, 3.24%), and *Gut* (3,155, 3.05%).

There is a concurrence of *Neurogastroenterology and motility: the official journal of the European Gastrointestinal Motility Society*, *World journal of gastroenterology*, *Alimentary pharmacology and therapeutics*, *The American journal of gastroenterology*, *Clinical gastroenterology and hepatology: the official clinical practice journal of the American Gastroenterological Association*, *Digestive diseases and sciences*, *Gastroenterology*, and *Journal of gastroenterology and hepatology* in the prolific journals and highly co-cited ones.

### Co-cited References and References With Citati on Bursts

In the 4,092 IBS publications, there were 1,386 references co-cited. Table 4 provides a list of the top 10 co-cited references. Of the eleven documents, five were published in *Gastroenterology*, two were published in *The New England journal of medicine*, one was published in *Clinical gastroenterology and hepatology: the official clinical practice journal of the American Gastroenterological Association*, one was published in *JAMA*, one was published in *The American journal of gastroenterology*, and the last one was from *Nature reviews. Disease primers*. Among them, Mearin et al. (24) published an article, entitled “*Bowel Disorders*” in *Gastroenterology*, which was the most frequently co-cited and ranked first (425), followed by “*Functional bowel disorders*”, written by Longstreth et al. (7) in *Gastroenterology* (266), “*Global prevalence of and risk factors for irritable bowel syndrome: a meta-analysis*,” authored by Lovell and Ford et al. (3) in *Clinical gastroenterology and hepatology: the official clinical practice journal of the American Gastroenterological Association* (246), and “*Functional Gastrointestinal Disorders: History, Pathophysiology, Clinical Features and Rome IV*,” published by *Drossman* (25) in *Gastroenterology* (205).

**TABLE 4:** Top 10 co-cited references in IBS research.

Rank	Reference	Citation	Year
1	Bowel disorders	425	2016
2	Functional bowel disorders	266	2006
3	Global prevalence of and risk factors for irritable bowel syndrome: a meta-analysis	246	2012
4	Functional gastrointestinal disorders: History, pathophysiology, clinical features and rome IV	205	2016
5	Irritable bowel syndrome: a clinical review	202	2015
6	An evidence-based position statement on the management of irritable bowel syndrome	173	2009
7	Irritable bowel syndrome	167	2016
7	The epidemiology of irritable bowel syndrome	167	2014
8	A diet low in FODMAPs reduces symptoms of irritable bowel syndrome	166	2014
9	Rifaximin therapy for patients with irritable bowel syndrome without constipation	151	2011
10	Irritable bowel syndrome	139	2017

As shown in Table 5, the highest-ranked co-cited references by the betweenness centrality value were published from 2008 to 2019. Of the nine references, two were published in *Gut*, two were published in *Alimentary pharmacology and therapeutics*, two were published in *The American journal of gastroenterology*, one was published in *World journal of gastroenterology*, and the other two were from *Gastroenterology* and *Gut*, respectively.

**TABLE 5:** Top 5 co-cited references with the highest betweenness centrality in IBS research.

Rank	Reference	Centrality	Year
1	A randomized trial of ondansetron for the treatment of irritable bowel syndrome with diarrhea	0.24	2014
2	American college of gastroenterology monograph on the management of irritable bowel syndrome and chronic idiopathic constipation	0.19	2018
3	Randomized clinical trial: pregabalin vs. placebo for irritable bowel syndrome	0.17	2019
4	Diet low in FODMAPs reduces symptoms of irritable bowel syndrome as well as traditional dietary advice: a randomized controlled trial	0.16	2013
4	Fecal microbiota composition and host-microbe cross-talk following gastroenteritis and in postinfectious irritable bowel syndrome	0.16	2017
4	Clinical trial: Asimadoline in the treatment of patients with irritable bowel syndrome	0.16	2008
5	Linaclotide for irritable bowel syndrome with constipation: a 26-week, randomized, double-blind, placebo-controlled trial to evaluate efficacy and safety	0.15	2012
5	Effectiveness of probiotics in irritable bowel syndrome: Updated systematic review with meta-analysis	0.15	2015
5	High risk of post-infectious irritable bowel syndrome in patients with Clostridium difficile infection	0.15	2016

**FIGURE 5:**
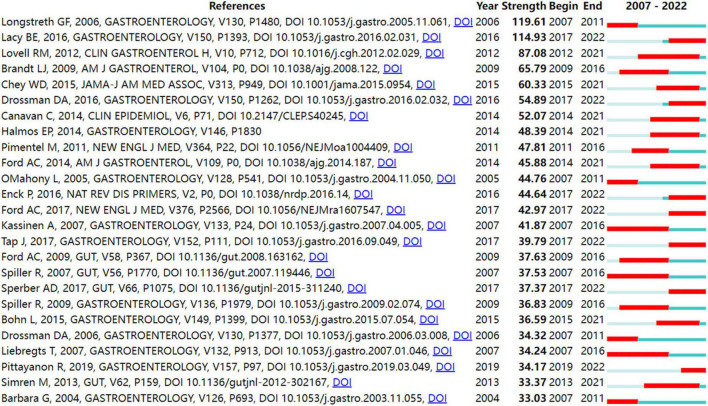
Top 25 references with strong citation bursts in IBS research.

In order to identify literature that has received a lot of attention from peers, the burst detection strategy was applied to publications cited at an increasingly fast rate. In Figure 5, strong citation bursts for 25 references are shown. Year denotes when the article was published. Strength represents the citation strength. The length of the line corresponds to the period from 2007 to 2022, in which the red segment indicates the time interval of citation bursts. The strongest citation burst was the article entitled “*Functional bowel disorders*” published in *Gastroenterology* by Longstreth et al. (7) with a citation burst lasting from 2007 to 2011 (119.61), followed by “*Bowel Disorders*” published by Mearin et al. (24) with a citation burst spanning from 2017 to 2022 (114.93), and “*Global prevalence of and risk factors for irritable bowel syndrome: a meta-analysis*,” published in *Clinical gastroenterology and hepatology: the official clinical practice journal of the American Gastroenterological Association* by Lovell and Ford et al. (3), which showed a citation burst from 2012 to 2021 (87.08). Those references whose citation bursts ended in 2021 or later deserve special consideration (2, 3, 24–34).

### Keywords Analysis

Keyword co-occurrence analysis is derived from the concept of citation coupling as well as co-citation in bibliometrics (35, 36). That is, when two keywords that reflect the core research contents of an article appear in the same document, it is considered that there exist the relationship between the two terms. The higher the number of co-occurrences of two terms, the closer their relationship is. A map of keywords co-occurrence is generated based on the frequency of appearance for paired keywords. One of the common methods of identifying hot topics in bibliometrics was co-occurrence analysis of keywords. In the present study, keywords were extracted from 4,092 publications. After excluding irrelevant keywords and merging those with the same semantic meaning, 773 keywords were identified.

**FIGURE 6:**
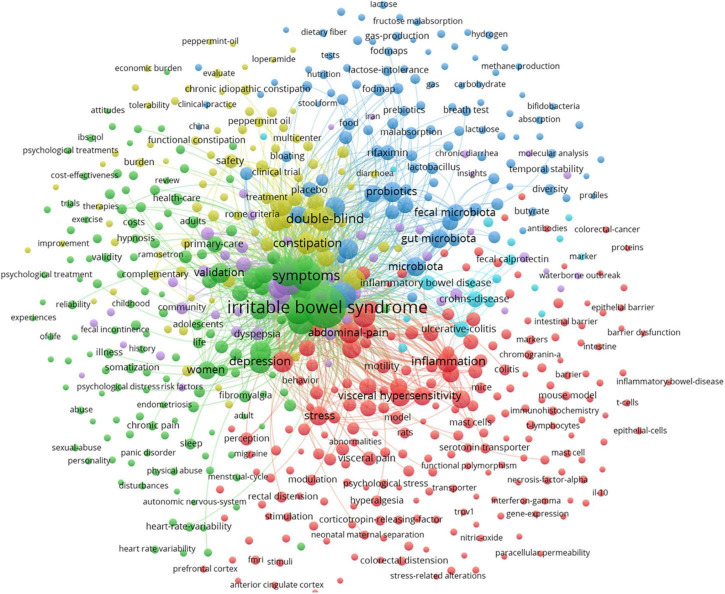
Map of keyword clustering with a minimum of 5 occurrences in IBS.

Figure 6 shows the map of keywords with highly co-occurrence frequencies that VOSviewer analyzed. Keywords were stratified into four clusters: clinical trials related to IBS (green cluster), post-infectious IBS (purple cluster), the role of the altered composition of intestinal microbiota in IBS (dark blue cluster), pathophysiological mechanisms of IBS (red cluster), IBS or IBS-like symptoms (light blue cluster), and pharmacological and non-pharmacological treatments for IBS (yellow cluster).

**FIGURE 7:**
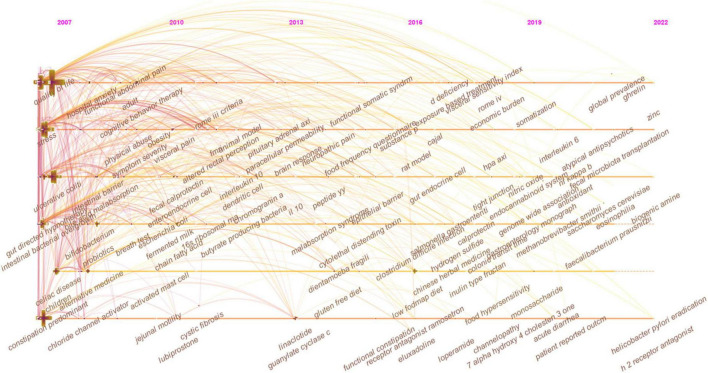
The timeline view of keywords in IBS research.

In Figure 7, the keywords co-occurrence was visualized in chronologic order. The year placed at the top of the view corresponds to the earliest year when each keyword appeared. Each node in the map represents a keyword. Co-occurrences of keywords are represented by the links.

**FIGURE 8:**
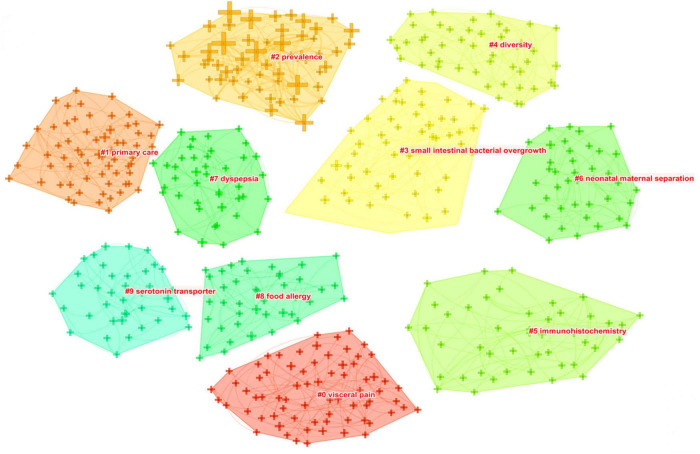
The keyword clustering knowledge map of IBS research.

As shown in Figure 8, closely related keywords were grouped into different clusters. A cluster is assigned a tag number, and the smaller the number, the more keywords comprise the cluster. The following 10 blocks were presented: #0 visceral pain; #1 primary care; #2 prevalence; #3 small intestinal bacterial overgrowth; #4 diversity, #5 immunohistochemistry; #6 neonatal maternal separation; #7 dyspepsia; #8 food allergy; and #9 serotonin transporter.

Table 6 presents the meaningful keywords with high frequency in IBS research. The most frequent keywords were symptom (1,020, 0.01), clinical trial (888, 0.1), quality of life (854, 0.01), epidemiology (819, 0.05), pain (566, 0), gut microbiota (518, 0.05), management (431, 0), hypersensitivity (424, 0.02), efficacy (297, 0.01), and constipation predominant IBS (276, 0.02).

**TABLE 6:** Top 20 keywords with the highest count in IBS research.

Rank	Keywords	Count	Centrality	Rank	Keywords	Count	Centrality
1	Symptom	1,020	0.01	11	Depression	256	0.03
2	Clinical trial	888	0.1	12	Risk factor	246	0.01
3	Quality of life	854	0.01	13	Stress	243	0.01
4	Epidemiology	819	0.05	14	Intestinal inflammation	238	0.01
5	Pain	566	0	15	Population	232	0
6	Gut microbiota	518	0.05	16	Anxiety	228	0
7	Management	431	0	17	Meta-analysis	213	0
8	Hypersensitivity	424	0.02	18	Diarrhea predominant IBS	211	0.01
9	Efficacy	297	0.01	19	Women	180	0
10	Constipation predominant IBS	276	0.02	20	Mast cell	179	0.01
10	Diagnosis	276	0				

**FIGURE 9:**
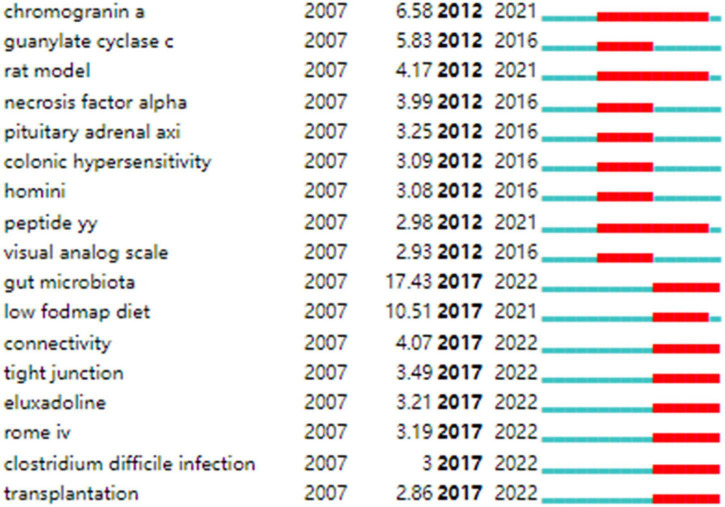
Keywords with strong citation bursts in IBS research.

Strong citation bursts are considered indicators of research frontiers within a particular period of time since the number of citations and occurrences of those terms have surged (19). Figure 9 shows the keywords with strong citation bursts. Some of them exhibited ongoing strong citation bursts, including chromogranin A, rat model, peptide YY (PYY), and gut microbiota, etc.

## Discussion

### General Information

The number of academic publications is an important reflection of research activity. As shown in Figure 1, a total of 2,039 documents were published from 2016 to 2021, a high-yield and rapid growth stage in the field, which has increased significantly compared with the previous 6 years, with 1,500 documents, from 2010 to 2015. Accordingly, it seems possible that the field is about to enter its golden period in the next few years.

Table 1 shows that the highest yielding countries are mostly based in Europe (England, Sweden, Italy, Germany, and France), Asia (China, South Korea, Japan), and North America (the United States and Canada). Any subject with a betweenness centrality value surpassing 0.1 is considered influential in the network. Thus, the highly productive countries in Europe and North America contributed the most impactful research in the field of IBS. Among the high-yield institutions, the top three ranked institutions were in the United States, while there was only one research institution in Asia (Iran). In addition, American and Swedish institutions, including Mayo Clin, Univ N Carolina, Univ Gothenburg, and Univ Washington were prominent in the field with considerable academic influence, given their high volume of publications as well as a high betweenness centrality value. Therefore, the United States and Sweden dominated in research quality and productivity; however, research capacities in Asian regions were generally weak. In the United States, for example, research capacity is likely related to overwhelming support in terms of research, the diversity of researchers with an interest in this field, a wealth of environments well-equipped for research, and the greater availability of a well-trained workforce. In addition, due to the strength of the economy of the United States, significant financial resources are made available to researchers, and scientists enjoy enhanced mobility (37, 38). Functional gastrointestinal disorders such as IBS are linked to dysbiosis, and the symptoms triggered is often caused by episodes that affect the microbiome in an environment where the emotional context and enteric nervous system are in synergy (39). Further, in 2013, the United States launched an innovative program on the gut microbiota-brain axis (40), which also has led to a surge in publications related to IBS.

As shown in Figure 2, in general, collaboration exerts positive effects on scientific output, and cooperative research results are of high scientific quality with high academic impacts, particularly those related to transnational collaboration. North American and European countries, which played an influential role in the IBS research, developed cooperative partnerships worldwide. However, collaborations in Asian countries tended to be intra-continental phenomena. It is possible that scientific advances in IBS research in Asian countries were plagued by less transnational cooperation and academic exchange. Notably, Canada and Ireland, which were detected with strong bursts, revealed high scholarly activity over a brief period.

In Figure 3, collaborations and partnerships among institutions mostly occurred within North America and Europe. Even though some Asian countries have contributed substantially to publications counts, they have not formed a cooperative network, which further confirms that IBS research in Asia lacked intercontinental collaboration. Teheran Univ Med Sci, Univ Calif Los Angeles, Mayo Clin, Univ Washington, McMaster Univ, Univ Manchester, and Cedars Sinai Med Ctr, which exhibited strong bursts, witnessed a large increase in recent publications.

In Table 2 and Figure 4, the productive authors were mainly from European and North American countries. Swedish and American researchers wielded major influence in IBS research, which also demonstrates the outstanding performance and leading roles of the United States and Sweden in the field. Instead, the academic impacts of Asian scholars were minor. Besides, the overall cooperation and communication still centered on European and American scholars. Hence, Asian nations, including China, South Korea, Japan, India, and Iran, are urged to follow the international pattern of fostering scientific cooperation while raising scientific output, which is directly linked to greater research quality and fortified scientific capability. In fact, a collaborative research project might lead to the participation of experts from various fields, which has been interpreted as positive evidence regarding its impact on the quality of research. As shown in Table 2, these prolific gastroenterologists who are also likely to initiate collaborations and in most cases provide the central funding or resource support in their community clusters, have interests in neurology, nutrition, and endocrinology (e.g., ALEXANDER C FORD, EMERAN A MAYER, MAGDY ELSALHY, and others). Moreover, the highest-ranked scholar by the betweenness centrality value was MAGNUS SIMREN, indicating that his academic attainments earned him great credibility among peers and had considerable influence in the field. Scholarly contributions from EMERAN A MAYER also occupied an eminent position.

According to Table 3, IBS research has been published largely in journals from Western countries that specialize in gastroenterology. Studies of high quality and well-designed design are the evidence base for IBS research, as the top prolific journals are typically found in Q1 or Q2. Journals with high co-citations are referred to as mainstream journals, to which researchers are dedicating great attention. Likewise, highly co-cited journals were issued in Western countries, which were classified as Q1 or Q2. This finding enhances the perception of strengthening the construction of scholarly periodicals, especially in Asian nations, for the generation of high-quality scientific outcomes and the dissemination of knowledge in the IBS field. Moreover, *Neurogastroenterology and motility: the official journal of the European Gastrointestinal Motility Society*, *World journal of gastroenterology*, *Alimentary pharmacology and therapeutics*, *The American journal of gastroenterology*, *Clinical gastroenterology and hepatology: the official clinical practice journal of the American Gastroenterological Association*, *Digestive diseases and sciences*, *Gastroenterology*, and *Journal of gastroenterology and hepatology* were deemed core journals in the field with high publications and co-citations. In addition to serving as reliable references for IBS-related manuscripts, they can also be taken into consideration when submitting manuscripts.

### Knowledge Base

Co-cited references are publications that have been cited together by other publications, and are viewed as a knowledge base for a particular field of study. As shown in Table 4, most literature published in high-impact journals between 2006 and 2017 were reviews or articles describing the epidemiology, risk factors, diagnosis, clinical features, pathophysiology, and management of IBS (1, 3, 4, 7, 24–26, 30, 41). In addtion, Halmos et al. (27) published the eighth co-cited paper in *Gastroenterology* in 2014; this study showed that low fermentable oligosaccharides, disaccharides, monosaccharides, and polyols (FODMAP) diet (LFD) which consisted in reducing the intake of poorly absorbed short-chain carbohydrates, such as lactose or fructo-oligosaccharides, improved gastrointestinal symptoms in IBS. Another co-cited article was published in *The New England journal of medicine* by Pimentel et al. (42). The researchers conducted two large Phase III trials of rifaximin in patients with non-constipated IBS, which demonstrated significant relief from symptoms of IBS such as bloating, abdominal pain, and watery or loose stools.

In Table 5, the top 5 co-cited references with the highest betweenness centrality value, which were considered key in defining the intellectual base of IBS, revolved around (1) therapies that target visceral pain modulation, including 5-hydroxytryptamine_3_ (5-HT_3_) receptor antagonist (43), the blocker of α2δ subunit on voltage-dependent calcium channels (44), and peripheral diaryl acetamide kappa-opioid receptor agonist (45); (2) therapies that increase intestinal secretion for IBS-C, such as agonist of the guanylate cyclase C receptor (46); (3) treatment targeting the intestinal microbiota (47); (4) non-pharmacological measure such as dietary modifications (32); (4) characterization of the intestinal microbiota in especially post-infectious IBS (48); and (5) risk factors for post-infectious IBS (49).

As can be seen in Table 6, the extant studies included in the analysis have primarily addressed IBS-C and IBS-D. The potential explanation for this trend may be that these subtypes are more prevalent. In a recent meta-analysis involving 6,756 participants, it has been reported that, using the Rome IV criteria, the global prevalence for IBS-D is 1.4%, followed by 1.3% for IBS-C, 1.1% for IBS-M, and 0.5% for IBS-U (50). In spite of the use of the Bristol stool form scale in Rome IV to categorize patients with IBS into subtypes, which results in a lower proportion of patients meeting the criteria for IBS-M or IBS-U, these individuals still comprised more than one third of the patients with IBS according to this meta-analysis (50). At present, however, there are no licensed therapeutics for use in these patients, which represents a significant unmet need. Therefore, enhanced research is needed.

Additionally, from Figure 7, in which “depressive symptoms,” “psychological distress,” “FODMAP,” “dysbiosis,” “microbiota,” “visceral hypersensitivity,” “functional magnetic resonance imaging (fMRI),” and “cortex,” are included in each cluster, this demonstrates that there has been a proliferation of research into brain-gut-microbiota (BGM) axis within global IBS research. We can infer that the IBS field will undergo a paradigm shift with the involvement of experts in psychiatry, neurology, microbiology, nutrition, and imaging.

### Hot Topics and Frontiers

Figure 5 shows the top 25 references with the strongest bursts of citations, whose research topics scholars followed closely over the past fifteen years. Among them, fourteen references (2, 3, 24–34) whose citation bursts continued to 2021 or later have attracted considerable interest from the scientific community, thus reflecting the hot topics and emerging trends in IBS research.

In Figure 7, the evolution of research topics was identified. In the early years from 2007 to 2013, IBS research began to focus on (1) overlap syndrome; (2) 16S ribosomal RNA gene sequencing; (2) fMRI, glucose breath test, and lactulose breath test; (3) IBS-like symptoms; (4) fibromyalgia, menstrual cycle, endometriosis, and chronic fatigue syndrome; (5) acute gastroenteritis, ischemic colitis, antibiotic-associated diarrhea, Inflammatory bowel disease (IBD), and celiac disease; (6) idiopathic constipation and obstructed defecation syndrome; (7) somatization, sexual abuse, physical abuse, and post-traumatic stress disorder; (8) gluten-free diet; (9) cingulate cortex, dorsal horn neurons, and prefrontal cortex; (10) serine protease activity, lactoferrin, and short chain fatty acids (SCFAs); (11) colonic fermentation, hydrogen sulfide, and methane production; (12) colonic hypersensitivity, altered rectal perception, allodynia, and neuropathic pain; (13) intestinal bacterial overgrowth; (14) butyrate-producing bacteria, lactic acid bacteria, *Escherichia coli*, *Blastocystis hominis*, *Lactobacillus rhamnosus* GG, *Lactobacillus reuteri*, and *Lactobacillus plantarum 299v*; (15) brain-gut axis and hypothalamic-pituitary-adrenal axis; (16) corticotropin-releasing hormone and cortisol; (17) PYY, cholecystokinin, and glutamine; (18) neonatal maternal separation; (19) enteroendocrine cells (EECs), dendritic cells, and T lymphocytes; (20) 5-hydroxytryptamine (5-HT) transporter, cannabinoid receptor, calcium channel, estrogen receptor-β, fibrosis transmembrane conductance regulator, and chloride channel activator; (21) E-cadherin; and (22) tumor necrosis factor-α (TNF-α), interferon-γ (IFN-γ), interleukin (IL)-10, and IL-1β.

From 2013 to 2016, the field focused on (1) early life stress, panic disorder, alexithymia, functional somatic syndrome; (2) collagenous colitis and malabsorption syndrome; (3) functional connectivity, anterior cingulate cortex, and catecholamine; (4) *Dientamoeba fragilis* and *Bifidobacterium infantis* 35624; (5) fecal microbiota transplantation (FMT); (6) visceral hyperalgesia and substance P; (7) interstitial cells of Cajal; (8) low-grade inflammation, paracellular permeability, and oxidative stress; and (9) δ opioid receptor and ion transport.

From 2016 to 2022, researchers turned to research on (1) food intake disorder, Brugada syndrome, and channelopathy; (2) Salmonella gastroenteritis and *Clostridium difficile* infection; (3) uroguanylin, fecal calprotectin, and heat-stable enterotoxin; (4) duodenal microbiome; (5) LFD; (6) water-avoidance stress; (7) bile acid metabolism; (8) *Faecalibacterium prausnitzii* (*F. prausnitzii*), *Akkermansia muciniphila*, *Methanobrevibacter smithii*, and *Saccharomyces cerevisiae*; (9) glucagon-like peptide 1, brain derived neurotrophic factor, ghrelin, and neuropeptide Y; (10) eosinophilia and mast cell; and (11) IL-6, nitric oxide, nuclear factor kappa B (NF-κB), and guanylyl cyclase C.

In addition, keywords with the ongoing strong citation bursts shown in Figure 9 were used to identify the hot issues within the field. Of these, gut microbiota is the keyword with the strongest citation burst. Given the hot topics are not separated, but influential and interrelated to each other. We discussed these key hot topics in IBS research under the most popular researched “gut microbiota” framework, highlighting their interrelated aspects as follows:

### The LFD

FODMAP are short-chain carbohydrates that are not readily absorbed in the small intestine, increasing water delivery into the lumen due to osmotic action causing diarrhea. FODMAPs act as a prebiotic for gas-producing bacteria, Clostridium, in the large intestine, increasing gas production (51). Luminal distension, in turn, is worsened. Among the metabolites fermented from FODMAP are SCFAs like butyrate, acetate, and propionate, as well as carbon dioxide and hydrogen. The presence of these metabolites might also affect microbial colonic environments and IBS symptoms (52, 53). The effects of butyrate on visceral sensitivity were demonstrated in healthy volunteers (54). Through stimulation of 5-HT release from the intestinal mucosa, SCFAs initiate high-amplitude propagated colonic contractions, accelerating intestinal transit (55).

Over the last decade, research has shown that the FODMAP-restricted diet may be a safe and effective dietary intervention (56). Several studies have been conducted to conclude that an LFD is effective in relieving overall IBS symptoms and behaves either with non-inferiority or superiority with respect to other comparators (27, 57, 58). The consumption of an LFD has been found to improve symptoms in more than half of IBS patients (59). However, these trials have been focused on showing their short-term effectiveness, and long-term studies still need to be carried out.

FODMAP restrictions may decrease levels of prebiotics, including fructo-oligosaccharides, galacto-oligosaccharides, and fibers, which are utilized by host microorganisms in a health-enhancing manner. In the end, this results in a reduced amount of highly beneficial bacteria and decreased production of SCFAs that are beneficial to colonocytes. Some studies have consistently reported the effect of an LFD leading to a reduction in *Bifidobactrium* (60–63), which is believed to be associated with a worse symptom profile, though no studies have yet investigated the detrimental effects of lower bifidobacteria resulting from LFD on long-term health. Twenty-seven IBS patients and six healthy participants were studied with an LFD or a typical Australian diet and researchers found *Clostridium Cluster IV* and *F. prausnitzii* levels were reduced in comparison to controls, with the latter known for anti-inflammatory properties due to its ability to produce butyrate, which regulates T helper 17 and T regulatory cells (62). An impaired level of *F. prausnitzii* could potentially harm the integrity of the intestinal mucous barrier, which results in dysbiotic microbes causing IBD (64). In a controlled, single-blind study with forty IBS patients (twenty on an LFD and twenty on a high FODMAP diet) for 3 weeks, researchers found that the LFD increased the richness and diversity of *Actinobacteria* (65). A recent review reports changes in gut microbiota composition after an LFD, such as a lower abundance of *Bifidobacterium* or Bifidobacteriaceae, Lactobacillaceae, Propionibacteriaceae, *Clostridium cluster IV*, *F. prausnitzii*, and an increased abundance of *Bilophila wadsworthia*, Clostridiales family XIII incertae sedis, and *Porphyromonas IV*.

In conclusion, mixed results were found in research conducted on the effects of LFD on gut microbiota and its metabolites. Inconsistencies between studies may be related to heterogeneity in LFD study designs, and different sample collection, storage, and analysis methodologies. In addition, feces analysis, however, does not reliably present the actual picture of the gastrointestinal tract. Metagenomics, transcriptomics, proteomics, and metabolomics can be more informative.

Another challenge with LFD is that only 50% of IBS patients report symptomatic improvement on an LFD. In an effort to optimize patient selection most likely to respond to the LFD, and to avoid unnecessary dietary restrictions in those less likely to respond, the potential cause of non-response is being investigated in greater detail. The question of whether baseline colonization of microbiota can predict symptomatic response to the diet is receiving increasing attention. In a clinical trial, thirty-one patients with IBS were randomly assigned to follow the LFD and thirty patients to follow National Institute for Health and Care Excellence dietary advice, and over 4 weeks, researchers found the dysbiosis index was higher in non-responders to the diet than in responders (63). Among responders, *Bacteroides stercoris*, *Pseudomonas*, *Acinetobacter*, *Desulfitispora*, *Parabacteroides*, *Bacillus*, *Salmonella* (*Citrobacter*, *Cronobacter*, *Enterobacter*), *Corea*, *Ruminococcus gnavus*, *Clostridium*, Firmicutes (*Clostridia*), and *Streptococcus* were lower at baseline (63).

In another study, 61 IBS adult patients followed the LFD for 4 weeks, with 52% of responders having different microbial composition at baseline when compared with 48% of non-responders (66). *Bacteroides fragilis*, *Acinetobacter*, *Ruminiclostridium*, *Streptococcus*, and *Eubacterium* were revealed to be higher in the responder group compared to the non-responder group and Clostridia/Negativicutes/Bacilli, *Actinomycetales*, Anaerotruncus, Clostridiales and *Shigella*/*Escherichia* were found to be lower in responders than non-responders at baseline (66). In both studies, the results regarding microbiota differences were noteworthy; it turned out that the results differed even though the intervention, selection criteria, and microbiota test were the same. Only one genus, *Streptococcus* was identified common in the studies but revealed opposing trends, indicating there is certainly much yet to learn about how symptom response to an LFD could be predicted by fecal bacterial profiles. In addition, fecal volatile organic compounds may serve as predictors of response to the LFD (67). Hence, gaps of interest include a deeper understanding of how an LFD affects the gut microbiota and research into diagnostic indicators such as bacterial markers and fecal metabolites to help to identify those likely to benefit from this specific intervention.

### Enteroendocrine Cells

It has been suggested that cellular components in the gastrointestinal mucosa contribute to IBS pathogenesis; much attention has been devoted to EECs now. The colonic glands are defined by the presence of serotonin-containing (enterochromaffin) cells, peptide YY (PYY)-, oxyntomodulin (enteroglucagon)-containing L cells, pancreatic polypeptide (PP)- and somatostatin-producing cells (68). Kyösola et al. (69) and Verity et al. (70) were among the first to study EECs in the intestinal mucosa of patients with IBS who reported increased numbers of EECs in rectal biopsies from these patients. However, this topic that has felt static for years begins to move.

Chromogranin A, a common marker for EECs, is a granin secreted from secretory granules of all the different types of EECs (71, 72). Patients with IBS have a lower density of chromogranin A in their duodenum and colon than healthy subjects, suggesting that their EECs are generally less dense (73). These abnormalities probably contribute to IBS pathophysiology because low-density EECs are characterized by subsequent low levels of certain hormones, and dysmotility of the gut, visceral hypersensitivity, and abnormal secretion may result from this in patients with IBS (74). Low cell densities of Musashi 1 (a marker for stem cells and their early progenitors) and neurogenin 3 (a marker for EEC progenitor) in the small and large intestines of IBS patients indicate that intestinal stem cells are low in clonogenic activity and differentiate slowly into endocrine cells (75, 76). Thus, it is proposed that the abnormal behavior of stem cells remains a possible cause of the low density of EECs (77).

Intestinal stem cells are a possible avenue through which factors contributing to the pathophysiology of IBS exert their effects. Specifically, the diet we consume is thought to act as a prebiotic, therefore stimulating certain species of bacteria to grow. In turn, the bacteria ferment the diet, releasing by-products that affect the stem cells and their progeny in a way that reduces their numbers and causes a low differentiation into endocrine cells, which finally results in low density in EECs and the development of IBS symptoms. However, gastrointestinal EECs interact and communicate with each other in complicated ways; it is reported that the higher densities of gastrin-producing cells and lower somatostatin-producing cells observed in the antrum of IBS patients cannot be explained by abnormal stem cells like those that are seen in the small and large intestines, given that the densities of Musashi-1-positive cells don’t differ between IBS patients and healthy controls in the stomach (78).

Furthermore, it has been established that diet interacts with EECs. This has been supported by the finding that in the stomach and colon of IBS patients, EECs detected by chromogranin A increase toward the values seen in healthy controls following an LFD, probably due to the changes in gastrin-, enterochromaffin-, ghrelin-, and somatostatin-secreting cells in the stomach and enterochromaffin cells and PYY containing L-cells in the colon (79–82).

Moreover, the gut microbiota is able to interact with EECs. FMT is shown to affect the densities of EECs in the duodenum and colon (83, 84). To investigate the mechanisms behind the restoration of EECs after receiving FMT, in a study by Mazzawi et al. (85), patients reported improvements in their IBS symptoms in parallel with changes in their EECs density 3 weeks after FMT. In fact, the changes in the density of EECs do not appear to be caused by an alteration in the stem cells or their early progenitors, rather they may be due to changes in the differentiation progeny, as observed by neurogenin 3 (85).

PYY which has been captured with a strong citation burst in our study is a hotly researched gut-derived hormone in IBS pathophysiology. This peptide along with enteroglucagon and glucagon-like peptide 1 is co-produced from L cells. The density of EECs, including PYY cells, in the colon and rectum, are lower in IBS patients when compared to healthy controls (86, 87). In this way, the presence of low amounts of PYY and low densities of PYY cells in the large intestine will impair the release of PYY, contributing to the dysmotility that is associated with IBS in that this hormone inhibits gastric and pancreatic secretion, delays the emptying of the stomach, and increases water and electrolyte absorption (88). Moreover, inferred from the fact that PYY modulates 5-HT release, which regulates visceral sensitivity, the low PYY concentration could indirectly contribute to IBS symptoms of visceral hypersensitivity (89). It has been reported that the consumption of LFD increases the PYY cell density to the normal level in IBS patients and improves the symptoms of IBS as well (82); in a similar manner, SCFAs, one of the fermentation products of intestinal microbes, have been reported to promote gene expression and stimulate the production of PYY (90). Hence, it is possible to restore PYY abnormalities by modifying the diet or the microbiota in IBS, and a PYY receptor stimulator could also be helpful for the treatment of IBS.

Taken together, the optimism in research on the links between diet, gut microbiota, stem cells, and EECs permits us to believe that the knowledge of stem cells and EECs (with hormones) will lead to their use in therapeutics and help to elucidate the mechanisms underlying improved symptoms and non-response following FMT and diet modification.

### Tight Junctions

While providing nutrients and water to the body, the intestinal barrier protects internal organs from bacteria, luminal antigens, and luminal pro-inflammatory factors (91). In several disease conditions, the intestinal barrier dysfunction causes bacteria, endotoxins, and other inflammatory mediators to proliferate. In the case of IBS, increased intestinal permeability was highly variable, with 2–62% showing increased permeability compared to 0–15% in controls (92). The access of noxious substances to submucosa is largely prevented by a network of tight junctions (TJs), adherens junctions, and desmosomes within the intestinal epithelium. A physiological condition allows only water and electrolytes to penetrate the epithelium on the paracellular level. Paracellular permeability is regulated principally by TJs, a network of proteins found at the apex of epithelial lateral membranes, including claudins (CLDN), occludin, junctional adhesion molecule-A, zonula occludens (ZO), etc. A loss of TJs can allow entry of antigenic macromolecules, lipids, peptides from microbes, and even microbes through the epithelium (93). As a result, the mucosal immune system is over-stimulated, which has been linked to visceral hypersensitivity and symptom generation in IBS.

In IBS, the mechanisms responsible for modulating TJ expression and assembly are complex. It has been suggested that the intestinal microbial changes are a potential driver. When compared with conventional mice, CLDN-1 and occludin levels were higher in germ-free mice with lower paracellular uptake of a standard probe (94). Based on this, it appears that commensal microbiota affects colonic TJ proteins and paracellular permeability. In germ-free rodents, De Palma et al. (95) examined the role of the microbiome in regulating intestinal permeability and found that the colonic barrier was disrupted following gavage of fecal slurry from IBS-D. Studies suggest that patients with IBS, particularly those with post-infectious IBS (PI-IBS), exhibit higher levels of fecal proteolytic activity (96). Dysbiosis-derived proteases contribute to TJ disruption in IBS through the activation of a protease-activated receptor pathway (97). A study reports that *Escherichia coli* Nissle 1917 can mitigate the increase in paracellular permeability associated with supernatants obtained from patients with IBS (98). Furthermore, the activation of toll-like receptor 4 (TLR4)/myeloid differentiation factor 88 (MyD88)/NF-κB pathway in IBS-D is linked to imbalanced inflammatory cytokine expression, finally affecting gastrointestinal motility, secretion, and re-absorption as well as increasing intestinal sensitivity (99, 100). More specifically, TLR4 and MyD88 are involved in pro-inflammatory signaling induced by bacterial lipopolysaccharide, whose activation may induce the expression of IFN-γ and TNF-α. In this way, intestinal epithelial barrier function is compromised by IFN-γ and TNF-α, which regulate the organization of several TJ proteins, such as ZO-1, CLDN-1, CLDN-4, and occludin (101). Therefore, blocking LPS-mediated signaling can be beneficial in protecting against the disruption of gut barriers. The TLR4-MyD88-transforming growth factor β-activated kinase1-NF-kB pathway is induced by wogonin to suppress inflammatory response and the down-regulation of CLDN-1 and ZO-1 in Caco-2 cells (102). IBS-D rats treated with QingHuaZhiXie prescription, a Chinese herbal compound prescription, showed that occludin, CLDN-1 and ZO-1 expression were restored in colon tissue by inhibiting the TLR4/MyD88/NF-κB pathway, which is accompanied by the improved symptoms of diarrhea and intestinal hypersensitivity (103).

In addition to the role of bacteria and their structural components in regulating TJ proteins, there may be different ways in which the microbial metabolites play a role. Butyrate, for instance, stimulates adenosine monophosphate-activated protein kinase, resulting in the assembly of TJ proteins which are important for intestinal barrier integrity repair (104). In response to sodium butyrate treatment, the motif-specific promoter region of CLDN-1 interacts with transcription factor specificity protein 1 to increase the transcription of CLDN-1 (105). It has been demonstrated that 6-formylindolo (3,2-b) carbazole, a tryptophan ligand, acts by activating the aryl hydrocarbon receptor and prevents TNF-α/IFN-γ-induced decrease in transepithelial electrical resistance and disruption of TJ proteins (106). Furthermore, polyamines, bile acid metabolites, conjugated fatty acids, and polyphenolic derivatives are examples of microbial metabolites that significantly affect TJ proteins (101).

Increased intestinal permeability has been observed in 37–62% of patients with IBS-D and 16–50% of patients with PI-IBS (92). IBS-C studies, however, showed the same level of permeability as controls (92). The rectosigmoid and descending colon represent the most extensively studied parts of the bowel in IBS, where TJ proteins are still highly heterogeneous. In addition, microbiota abundance and composition are not only related to a particular region within the digestive system, but also to the place where they are sampled. Also, in IBS, remissions are mixed with periods of symptoms escalating. Hence, to identify microbial changes that are missed with cross-sectional sampling, longitudinal sampling strategies, multiple time-point samples, a post-intervention follow-up, and a washout period for cross-over studies are needed; similarly, to better understand target TJ proteins in IBS, it may prove beneficial to sample longitudinally different areas of the gut in the same volunteers.

Even though some promising findings have been made, the intricate relationship between altered microbiota composition with microbial metabolites and TJ proteins modulation should be further explored, particularly in IBS-D and PI-IBS subtypes.

### Neuroimaging and Gut Microbiota

Usually, IBS is a medical label used for medically unexplained gastrointestinal symptoms, but it may also reflect disturbances of the BGM axis (8). Since changes in the composition or functions of gut microbiota are known to affect human behavior and brain physiology, and dysbiosis is often presented by IBS patients, a great deal of attention has been paid to the role that gut microbiota play in this interaction (107). There is no adequate understanding of how gut microbiota signaling to the brain in humans works, but this process appears to be either modulated by microbial interactions with the host or diet, producing neuroactive compounds that can send signals to the brain *via* afferent vagal pathways or humoral channels or mediated by bacterial metabolites, which regulates immune function and cytokine production with downstream effects on brain functions through the regulation of neuroinflammation (108).

The use of neuroimaging as a non-invasive tool to explore the mechanisms of these pathways can be useful in addition to measuring the bioactives. In a new perspective on disorders of the BGM-axis, brain networks (brain connectome) and networks of gut cells and microbiota (gut connectome) are integrated, leading to a significant increase in studies that have combined gut microbiota and neuroimaging to investigate IBS pathophysiology (109).

Besides identifying structural and functional changes in specific brain regions, neuroimaging studies largely focus on brain connectivity using techniques such as diffusion MRI or fMRI recordings coupled with topological networks (110). The study by Labus et al. (111) examined the relationship between gut microbiota and brain structure among IBS patients. An increase in Clostridium members was significantly associated with a larger volume of subcortical areas (putamen, caudate nucleus, nucleus accumbens) and lower insula and prefrontal cortex volumes among the IBS participants (111). They also focused on Clostridiales, and examined its association with brain function and gastrointestinal sensorimotor function in another study (112). *Lachnospiraceae incertae sedis*, *Clostridium* XIVa and *Coprococcus*, all within the order Clostridiales, were found to be associated with gastrointestinal sensorimotor function in healthy controls, but not in IBS (112). Within the subcortical regions, *Clostridium* XIVa negatively correlated with putamen connectivity, as did Coprococcus and caudate nuclei, which were both related to improved gastrointestinal sensorimotor function (112). However, in IBS, there was a positive association between *Clostridium* XIVa and the putamen, the caudate nucleus, and the thalamus connectivity, suggesting that these subcortical areas may be affected by *Clostridium* XIVa and *Coprococcus*, and this may contribute to visceral hypersensitivity and pain in IBS (112). Osadchiy et al. (113) also reported on fecal metabolites and resting state fMRI. Histidine, glycine, glutamate, spermidine, and anserine variations were significantly correlated with changes in left dorsal part of the posterior cingulate gyrus to the left putamen (113). In addition, variations in histidine, tryptophan, uracil, 2-deoxyuridine, thymidine, and succinate were related to changes in the right superior frontal gyrus to right putamen. Aberrant tryptophan signaling may be responsible for this interaction in IBS patients (113). In fact, tryptophan is an important precursor of 5-HT. Several studies have suggested that tryptophan may be an essential amino acid in IBS because 5-HT is important for secretion, absorption, and intestinal transit, as well as mood, pain, and cognitive functions (114). Recently, Jacobs JP et al. (115) have shown that among IBS patients, as compared to non-responders, those who responded to cognitive behavioral therapy had increased levels of several members of the Clostridiales order as well as decreased levels of Bacteriodales. Responders showed a reduction in connectivity across multiple cortical networks including sensorimotor, default mode, salience, and emotion regulation networks following treatment, and these brain changes occurred in conjunction with a conversion to Bacteroides-predominant microbiota (115).

Microbiota together with non-invasive techniques that assess brain function, such as fMRI, have shed light on some aspects of the BGM-axis. The Bergen BGM-study (116), among others, provides an excellent example of multimodal and interdisciplinary clinical studies designed to verify directionality and causality in the BGM-axis in IBS.

### Fecal Microbiota Transplantation

FMT involves the application of a fecal solution from a healthy contributor into the gut of a receiver that is aimed at restoring a dysfunctional microbial composition to a healthy one, and so to improve the function of the gut microbiota. In addition to its commonly recommended use in *Clostridioides difficile* infection, FMT has also recently gained attention due to the strong evidence linking dysbiosis to IBS pathogenesis (117). No consensus exists regarding FMT procedure, different routes of FMT delivery (e.g., colonoscopy, nasogastric tube, enema, and oral capsules), the types of formulations (frozen, dried and fresh), and the number and type of donors were examined. Each of these modes of treatment has had varying degrees of clinical success.

According to a meta-analysis of 267 IBS patients, colonoscopic FMT treatment was effective, nasogastric tube treatment was marginally beneficial, and oral capsules failed to deliver benefits (118). The results of another study investigating the effectiveness of FMT *via* colonoscopy for the treatment of IBS patients with diarrhea or diarrhea and constipation showed that clinically significant improvement of symptoms was observed in 65% of patients undergoing FMT after 3 months, compared to 43% of control subjects receiving their own feces (119). In patients with frozen FMT, as opposed to fresh transplants, better results were obtained (119). In addition, other studies suggest that fresh or frozen donated stool may provide benefits, while capsulized FMT could be harmful (118, 120). El-Salhy et al. (121) conducted a study evaluating the effects of two different doses (30 and 60 g) of FMT. There was a response in 23.6% of the patients who received a placebo, 76.9% who got 30 g FMT, and 89.1% who got 60 g FMT, suggesting a dose-dependent response (121). Holvoet et al. (122) suggested a microbiota modulation strategy through FMT could benefit the subgroups with severe bloating and flatulence, in whom there is the most profound disruption to gut microbial composition. FMT also appears to be dependent on the donor, so having a superdonor available would be crucial to ensuring the success of treatment (123). In addition, having a greater diversity of microbes pre-FMT makes it more likely that the individual will respond positively to FMT (122). Increasing microbial diversity would seem counterintuitive if FMT succeeded; possibly other transplanted components were responsible for its efficacy.

A Danish trial by Halkjær et al. (124) presented results that conflicted with the reporting of beneficial effects. During this randomized, double-blind, and placebo-controlled trial, 52 patients with moderate-to-severe IBS were randomized to FMT capsules or placebo for 12 days, with a follow up of 6 months. Although FMT altered gut microbiota composition, patients in the placebo group experienced greater relief of symptoms after 3 months than those in the treatment group. The authors state that altered gut microbiota does not suffice to obtain clinical improvement in IBS (121). In this regard, it remains to be seen if altered microbiota composition, function, and abundance contribute to rather than result from IBS.

FMT has been accompanied by sparse long-term follow-up data in people with IBS, but it must be stressed that it is not without risks. A study found 20% of the FMT group experienced side effects vs. 2% of the autologous FMT group, including two patients with diverticulitis in the FMT group and none with diverticulitis in the patients with autologous feces (121). FMT, as currently practiced, is only investigated in a research setting, and the available data on its potential effects is not sufficient to make any conclusive conclusions. Before the FMT can be made available as an openly available treatment option, more large-scale clinical trials are necessary.

Based on the hot topics outlined, we know that there has been a marked improvement in knowledge regarding IBS in recent years, with an increasing understanding of the BGM axis as well as potentially effective therapeutic options. Since IBS is defined as one of disorders of the gut-brain interaction, psychological aspects of IBS require attention.

An estimated 44 and 25% of IBS patients seen in gastroenterology clinics with anxiety and depression constitute the significant cohort of psychiatric comorbidity, respectively (125). The proposed biopsychological model of IBS implies that gut microbiota influence anxiety and depression secondarily (bottom-up model) and that psychological factors themselves lead to gut microbiota reconfigurations (top-down model) (126). To date, however, the mechanisms underpinning these psychological comorbidities remain unresolved.

It is interesting to note that most IBS patients with comorbid anxiety and depression manifest gastrointestinal symptoms prior to presenting with psychiatric symptoms (127) and as described by our analysis, the current landscape of IBS research also appears to primarily focus on the bottom-up approach, with efforts to delineate the roles of gut microbiota in dysregulation of the hypothalamic-pituitary-adrenal axis, the crosstalk between gut microbiota and the host’s immune system (e.g., microbe-associated molecular patterns), the increased intestinal permeability in the setting of inflammation, and the involvement of microbial metabolites, such as SCFAs and neuroactive molecules, in gut-brain communication. The presence of neuroinflammation associated with low-grade intestinal and systemic inflammation in IBS seems to contribute to psychiatric comorbidity (128–130), despite there still being a limited understanding of the nature of this entity that sits at the intersection between IBS, depression, and anxiety. In fact, in few studies, gut microbiota signatures associated with psychiatric comorbidity in IBS have been evaluated (95, 131, 132). In these studies, at least, it appears likely that comorbid patients cluster differently from patients with IBS, depression, or anxiety alone. Research efforts should be intensified to uncover why certain microbiota alterations result in IBS in some cases while in other cases leading to IBS with psychological disorders.

It has also been established but recently further demonstrated in recent preclinical studies that stress, a major contributor to the development of IBS and depression in later life, can also affect the composition and function of the gut microbiota (133, 134). BGM axis is a bidirectional pathway; however, research on top-down hypothesis in this subset of patients appears to be lagging.

## Conclusion

In this study, based on the 4,092 documents on IBS research retrieved from the WOSCC from 2007 to 2022, we conducted a bibliometric analysis of the knowledge structure, active research topics as well as emerging trends of IBS research. Overall, scientific production showed an upward trend. The United States and Sweden remained dominant in the IBS field with a high number of publications, great scholarly impact, and broad collaboration network in terms of authorship, (intra- and inter-) nationally and institutionally. MAGNUS SIMREN was the predominant contributor to the field with a high academic impact. *Neurogastroenterology and motility: the official journal of the European Gastrointestinal Motility Society*, *World journal of gastroenterology*, *Alimentary pharmacology and therapeutics*, *The American journal of gastroenterology*, *Clinical gastroenterology and hepatology: the official clinical practice journal of the American Gastroenterological Association*, *Digestive diseases and sciences*, *Gastroenterology*, and *Journal of gastroenterology and hepatology* were deemed core journals in the field with high publications and co-citations. In addition, this study identified the LFD, EECs, tight junctions, neuroimaging and gut microbiota, and FMT as the research foci in the IBS field.

## Data Availability Statement

The original contributions presented in this study are included in the article/supplementary material, further inquiries can be directed to the corresponding author/s.

## Author Contributions

XT, FW, and BZ led the team and were responsible for all aspects of the project. TZ, XM, and WT contributed to the methods, data acquisition, results, and interpretation. WT, JZ, and YW participated in designing and writing the manuscript. TZ, BZ, WT, and JZ revised this manuscript critically for important intellectual content. XT gave final approval of the manuscript. All authors contributed to the article and approved the submitted version.

## Conflict of Interest

The authors declare that the research was conducted in the absence of any commercial or financial relationships that could be construed as a potential conflict of interest.

## Publisher’s Note

All claims expressed in this article are solely those of the authors and do not necessarily represent those of their affiliated organizations, or those of the publisher, the editors and the reviewers. Any product that may be evaluated in this article, or claim that may be made by its manufacturer, is not guaranteed or endorsed by the publisher.
